# The Impact of Protective Face Coverings on Acoustic Markers in Voice: A Systematic Review and Meta-Analysis

**DOI:** 10.3390/jcm12185922

**Published:** 2023-09-12

**Authors:** Ben Barsties v. Latoszek, Viktoria Jansen, Christopher R. Watts, Svetlana Hetjens

**Affiliations:** 1 Speech-Language Pathology, SRH University of Applied Health Sciences, 40210 Düsseldorf, Germany; 2 Harris College of Nursing & Health Sciences, Texas Christian University, Fort Worth, TX 76109, USA; 3 Department for Medical Statistics and Biomathematics, Medical Faculty Mannheim, University of Heidelberg, 68165 Mannheim, Germany

**Keywords:** respiratory protection masks, acoustics, corona pandemic, COVID-19, voice quality, dysphonia

## Abstract

Background: Wearing respiratory protective masks (RPMs) has become common worldwide, especially in healthcare settings, since the onset of the COVID-19 pandemic. Hypotheses have suggested that sound transmission could be limited by RPMs, which possibly affects the characteristics of acoustic energy and speech intelligibility. The objective of this study was to investigate the effect of RPMs on acoustic measurements through a systematic review with meta-analysis. Methods: Five database searches were conducted, ranging from their inception to August 2023, as well as a manual search. Cross-sectional studies were included that provided data on widely used gender-independent clinical acoustic voice quality measures (jitter, shimmer, HNR, CPPS, and AVQI) and habitual sound pressure level (SPL). Results: We found nine eligible research studies with a total of 422 participants who were compared both without masks and with different types of masks. All included studies focused on individuals with vocally healthy voices, while two of the studies also included those with voice disorders. The results from the meta-analysis were related to medical/surgical and FFP2/(K)N95 masks. None of the acoustic measurements showed significant differences between the absence and presence of masks (*p* > 0.05). When indirectly comparing both mask types, statistical significance was identified for parameters of jitter, HNR, CPPS and SPL (*p* < 0.001). Conclusions: The present meta-analysis indicates that certain types of RPMs have no significant influence on common voice quality parameters and SPL compared to recordings without masks. Nevertheless, it is plausible that significant differences in acoustic parameters might exist between different mask types. Consequently, it is advisable for the clinical practice to always use the same mask type when using RPMs to ensure high comparability and accuracy of measurement results.

## 1. Introduction

Multidimensional voice evaluation (e.g., visual analysis, auditory-perceptual judgment, aerodynamic analysis, acoustic analysis, and self-assessment [1]) is essential to determine the degree and type of dysphonia with individual voice complaints in patients with voice disorders. However, in order to provide a high measurement of accuracy to the individual measurement procedures on the one hand, but also to protect the investigator staff and the patients on the other hand, it is necessary to take the appropriate safety precautions. For example, during the coronavirus pandemic from 11 March 2020 to 5 May 2023, without a global emergency [2], the aim was to control and prevent all coronavirus-related diseases and mortality, particularly through hand hygiene, social distancing and nose-and-mouth-covering respiratory protective masks (RPMs). To avoid potential infection through respiratory droplets or airborne transmission, various RPMs such as medical/surgical or FFP2/(K)N95 masks were widely used in everyday life and clinical settings and are still in use today. Research conducted over the past three years has suggested the hypothesis that sound transmission is limited when passing through RPMs, which act as a low-pass filter, attenuating sound in the mid- and high-frequency ranges with consequences on perceptual speech intelligibility and acoustic properties of the voice [3,4,5]. Face masks might alter people’s perception of sound by reducing the perceived loudness and selectively removing certain sound components, particularly the high-frequency elements that are critical for a clear understanding of spoken language. This could be even more evident when a phone is used with a face mask, as the filtering of these important high frequencies is even more impaired, especially in special groups, such as individuals wearing cochlear implants or hearing aids or individuals with communication issues such as dysphonia and aphasia. Thus, the wearers of masks would speak louder, which, therefore, would lead to more effort and the development of vocal fatigue and a potential increase in vocal discomfort. Several systematic reviews or others without meta-analyses have attempted to summarize and examine acoustics, the effects on aerodynamic characteristics, self-reported characteristics of vocal effort and fatigue, vocal tract discomfort, and voice handicap index [6,7,8,9]. Overall, the results of these literature reviews did not unanimously show that the results of acoustic markers such as habitual intensity [6,8], formant frequencies of F2 and F3 [6], harmonics-to-noise-ratio [6,8] and mean spectral values in high-frequency levels (1000–8000 Hz) [6,7,8] may be influenced by wearing face masks; however, there is a lack of a clear statement of the expected distribution when an overall significant effect was statistically demonstrated across the single studies.

Acoustic measurements for voice quality and vocal function analyses have a main part in the clinical examination routine of laryngology, evaluating the degree and type of dysphonia. If this RPM effect significantly impacts acoustic voice analysis assessing voice quality parameters and vocal function parameters such as loudness/intensity, these effects must be taken into account in the recording procedure and analysis since they would influence measurement accuracy (e.g., threshold values). Therefore, the present study is, to our knowledge, the first study that investigated the effect of RPMs on the outcome of gender-independent acoustic parameters of voice quality and vocal function in a meta-analysis.

## 2. Materials and Methods

### 2.1. Data Sources and Searches

We utilized the reporting guideline provided by the Preferred Reporting Items for Systematic Reviews and Meta-Analyses (PRISMA) to systematically search five databases (MEDLINE, CENTRAL, LIVIVO, Speechbite, and Google Scholar) from their inception until 2 August 2023. A combination of different keywords such as “face mask”, “acoustic”, and “voice quality” was used, and a comprehensive list of these keywords can be found in Table S1 of the Supplementary Materials.

Potential articles were initially identified based on their titles and abstracts. Furthermore, a manual search of the grey literature sources was conducted. This involved examining the bibliographies of the included studies to identify additional relevant articles. The process of hand searching was carried out for scientific reports published in both German and English languages, and those included in the databases were considered for the meta-analysis.

### 2.2. Study Selection

The present study included cross-sectional studies that investigated the effect on acoustic parameters with and without RPMs (i.e., medical/surgical masks and FFP2/(K)N95). To minimize variation in specifications and reliability caused by the use of different acoustic software packages, we considered only studies that performed acoustic measurements with Praat (developed by Paul Boersma and David Weenink at the Institute of Phonetic Sciences, University of Amsterdam, The Netherlands: http://www.praat.org/ accessed on 13 August 2023). This meta-analysis included widely used quantitative acoustic measures from an internationally recognized set of gender-independent voice quality measurements encompassing key clinical parameters such as jitter (Jit%) [1], shimmer (Shim%) [1], harmonics-to-noise ratio (HNR) [10], smoothed cepstral peak prominence (CPPS) [10], and Acoustic Voice Quality Index (AVQI) [11]. Furthermore, habitual sound intensity level or sound pressure level (SPL) as a vocal function parameter and relevant for a voice diagnostic battery was also considered [1].

To be included in this meta-analysis, studies had to include at least one of these acoustic measures assessing habitual voice production of sustained vowel /a:/ (jitter, shimmer, HNR, CPPS, and sound intensity level) or the standardized concatenation of continuous speech and sustained vowel /a:/ for AVQI in voice evaluation. 

### 2.3. Risk of Bias Assessment

The risk of bias assessment of the included studies was determined using the checklist for cross-sectional studies of 11 items from the Agency for Healthcare Research and Quality (AHRQ) [12]. For a better interpretation, a high quality with a low risk of bias was assessed when ≥75% were answered with “yes”. A moderate risk of bias was present when 50% to 75% of items received a confirmation, and a high risk of bias was provided below 50% replies with “yes” for all items. Items marked with “unclear” reduces the total number of 11 items for the individual study.

### 2.4. Data Extraction

Two reviewers (B.B.v.L. and V.J.) extracted the data. Any discrepancies were resolved through discussion. Information collected from the selected studies included details such as article attributes (authors, publication year, journal, article title), study characteristics (research design, sample size, participants with voice disorders compared with vocally healthy individuals, acoustic data processing methodology, results with and without face masks), patient demographics (age and gender), and outcomes (jitter, shimmer, HNR, CPPS, AVQI, and SPL).

### 2.5. Statistics

The statistical analyses were conducted using MedCalc software (version 19.6) and SAS software (version 9.4).

First, the differences between the mean values of study parameters ${\bar{x}}_{without\;mask}-{\bar{x}}_{mask}$ and standard errors (SE) were calculated $\mathrm{SE}=\frac{(S_{1}+S_{2})/2}{\sqrt{n}}$.

Second, meta-analyses were performed using MedCalc software version 19.6 for the six parameters: Jit%, Shim%, CPPS, HNR, AVQI and SPL. For each parameter, the mean difference (MD) was calculated along with a 95% confidence interval (CI) for each individual study. To present the results of the meta-analyses visually, a forest plot was used. An I^2^ index was used to assess heterogeneity between studies included in the analysis. According to Higgins definition [13], I^2^ = 0%: there is no observed heterogeneity; I^2^ > 0% and ≤25%: there is insignificant heterogeneity; I^2^ > 25% and ≤50%: there is low heterogeneity; I^2^ > 50% and ≤75%: There is moderate heterogeneity; and I^2^ > 75%: there is high heterogeneity. Since in the meta-analysis there are differences in the characteristics of the population or in other factors, leading to heterogeneity or dissimilarity in the results, the random effect model was used so that heterogeneity between studies was accounted for. The weighting, according to DerSimonian and Laird [14], was used.

Third, an indirect comparison between medical/surgical masks and (K)N95/FFP2 was calculated. For this purpose, the two confidence intervals were compared using Welch’s *t*-test. A confidence interval is the estimate of the basic population and contains more information than a direct comparison. A *p*-value of <0.05 was considered statistically significant.

## 3. Results

### 3.1. Study Characteristics

**Figure 1:**
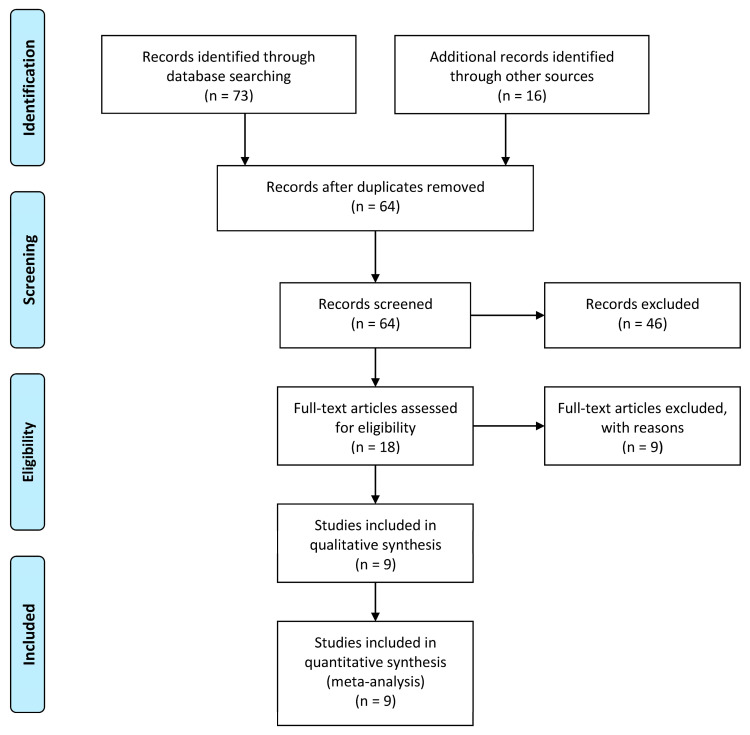
Prisma flow diagram.

During our searches, we encountered 64 unique papers (see Figure 1). Of these, nine studies met the criteria for inclusion in the current review. The study details of these selected papers can be obtained in Table 1 [15,16,17,18,19,20,21,22,23]. In total, 422 volunteers were investigated without and with different types of masks, ranging from seven to one hundred and fifty-nine participants in the studies. All studies included vocally healthy individuals, and two studies also included different types of voice disorders and severity degrees of dysphonia. The risk of bias assessment is shown in Table S2 of the Supplementary Materials, in which most cases have a moderate risk of bias, and in two cases [17,21], a low risk of bias was assessed.

### 3.2. Meta-Analysis

**Figure 2:**
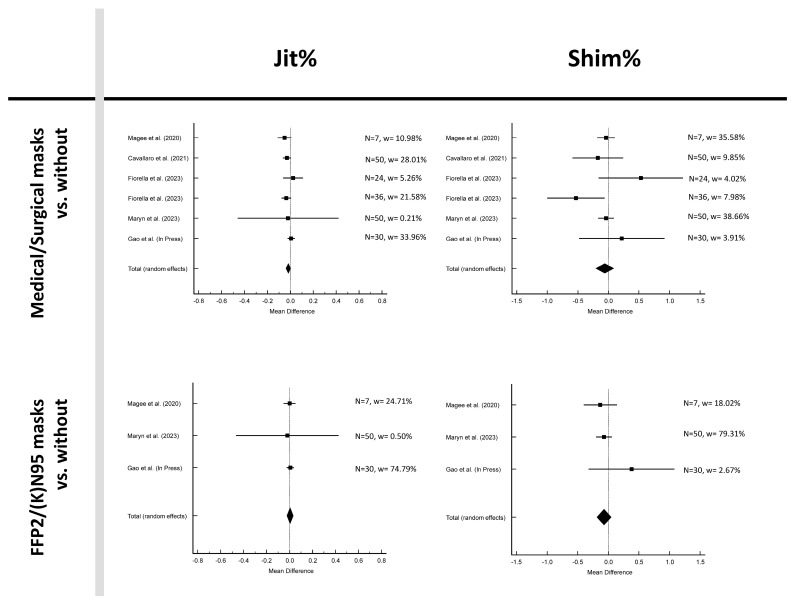
Forest plots of measurements Jit% and Shim% [15,16,20,21,23].

**Figure 3:**
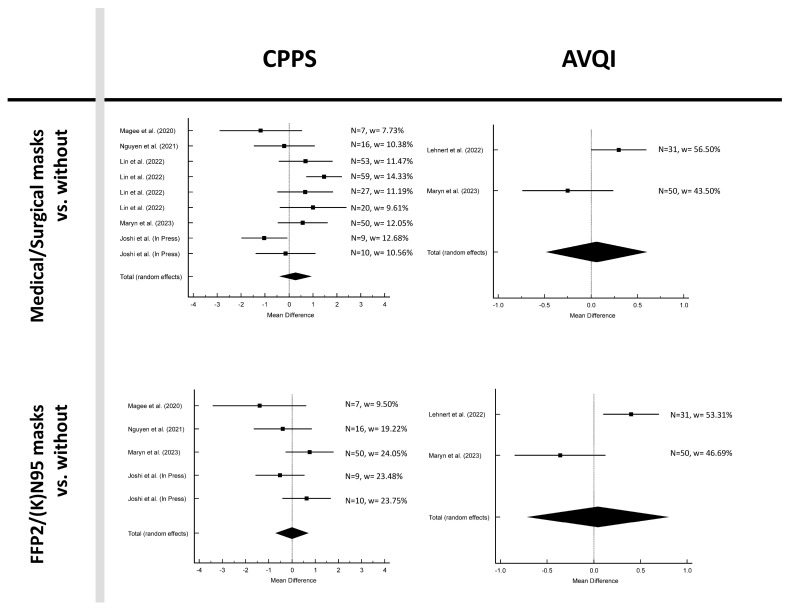
Forest plots of measurements CPPS and AVQI [15,17,18,19,21,22].

**Figure 4:**
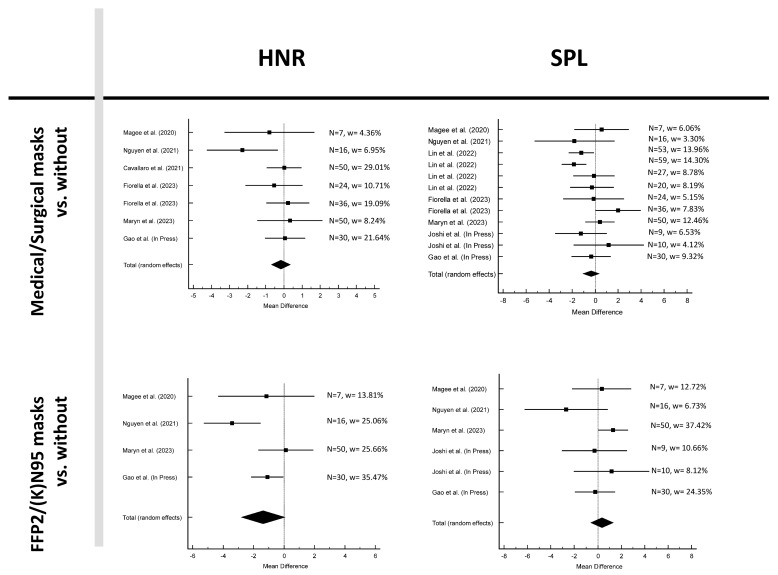
Forest plots of measurements HNR and SPL [15,16,17,18,20,21,22,23].

Figure 2, Figure 3 and Figure 4 and Table 2 show the results for the six parameters: Jit%, Shim%, CPPS, HNR, AVQI, and SPL. The mean difference between without mask and medical/surgical mask was negative for parameters Jit%, Shim%, HNR and SPL and positive for CPPS and AVQI. The findings from the meta-analysis, along with heterogeneity statistics and assessments of publication bias, revealed no publication bias in all parameters and no heterogeneity in Jit% and HNR, low heterogeneity in Shim% and SPL, and moderate heterogeneity in CPPS and AVQI.

In the analysis between without mask and FFP2/(K)N95, the mean difference was negative for the parameters Shim% and HNR and positive for Jit%, CPPS, AVQI, and SPL. No publication bias was present in all parameters. Furthermore, no heterogeneity was revealed in Jit%, Shim%, and SPI, low heterogeneity in CPPS, moderate heterogeneity in HNR, and high heterogeneity in AVQI was found.

None of the parameters were significant when comparing acoustic measurements with and without masks (*p* > 0.05; see Table 2).

### 3.3. Indirect Comparison between the Two Mask Types

The comparison between FFP2/(K)N95 and medical/surgical masks showed significance for the parameters Jit%, HNR, CPPS, and SPL (*p* < 0.001). The pooled difference of the Jit% parameter for medical/surgical mask was larger than FFP2/(K)N95 (MD (without mask–medical/surgical mask): −0.02 and MD (without mask–FFP2/(K)N95 mask): 0.01). For the parameter HNR, the pooled difference for FFP2/(K)N95 was larger than for medical/surgical mask (MD (without mask–medical/surgical mask): −0.17 and MD (without mask–FFP2/(K)N95): −1.37). For the parameter CPPS, the pooled difference for medical/surgical mask was larger than for FFP2/(K)N95 (MD (without mask–medical/surgical mask): 0.28 and MD (without mask–FFP2/(K)N95): 0.003). For the parameter SPL, the two signs were contradictory (MD (without mask–medical/surgical mask): −0.35 and MD (without mask–FFP2/(K)N95 mask): 0.36).

## 4. Discussion

The aim of this study was to examine how RPMs impact six gender-independent acoustic parameters connected to voice quality and vocal function among vocally healthy and voice-disordered individuals. For six acoustic parameters, there was no significant effect detected when comparing measurements with or without a RPM. However, there were differences between mask types, which led to recommendations of caution in the clinical routine when masks have to be used. The included publications had mostly a moderate risk of bias, while two of the nine studies revealed a low risk of bias. The heterogeneity ranged from no to high, but eight out of twelve analyses yielded no or low heterogeneity. Just one outcome presented a high heterogeneity for AVQI, which was twice investigated. There was no evidence of imprecision, publication bias, or indirectness.

The present meta-analysis was useful to assess an overall picture of the possible impact on the outcome of acoustic measurements based on RPMs. Although some systematic reviews and further single studies noted for HNR [6,8,17,23,24,25], Jitter [26], Shimmer [24,26], CPPS [18,21], SPL [6,8,18,26], and AVQI [21] significantly effects by RPMs, the present evaluation of this meta-analysis did not support these findings using the software Praat for the signal processing of the included acoustic measures. Further studies that were not included in the present meta-analysis (based on different signal processing methods of the acoustic parameters or other mask types) also concluded that for the same acoustic measures for vocally healthy and voice-disordered individuals, no significant differences between wearing a RPM or not [27,28,29]. Moreover, it must be taken into account that voice physiology and voice characteristics may differ between different speakers and between single or multiple voice recordings for consistency of sound measurements [30,31]. Although studies asked speakers to produce the same utterances while wearing a mask and while not wearing or changing masks, a fluctuation of voice is presented, at least to a small extent and should be controlled by the investigator groups. Due to minimal changes in the outcomes of the acoustic parameters between the recordings, a clinical significance could not be clearly observed. Although wearing masks for a prolonged period of time may cause observable self-perceived changes (e.g., breathing difficulties, increased effort of speaking, greater perception of symptoms of vocal fatigue and discomfort and changes in speaking behavior) in wearers [32,33,34,35], this influence remains unexplained in the present study, notwithstanding the fluctuations in multiple recordings of the voice analyses. To verify mask effects after prolonged wearing, it should be further investigated in future studies. Moreover, the possibility of individuals adjusting their speaking behavior when wearing masks is, in this context, also valuable to investigate and assess the potential impact of the validity of the acoustic measurements. Another confounding factor with a significant impact on the present results could be the recording hardware from the different studies. There are standards defined for instrumental assessments of voice recordings for vocal function to minimize bias and increase the comparability of studies [36]. A quick check revealed that some included studies could have deviations with regard to these recording standards [15,17,18,22].

The only significant effect demonstrated by the current meta-analysis was a measurable, significant difference in most of the included parameters based on mask type. Thus, it is recommended, for a clinical routine in laryngological practice, to always use the same mask type and not change. This will allow for comparability in the results between mask-wearing and without, and no systematic error in intra- or inter-individual comparison of patients’ recordings and their analyses is present. In real-world clinical practice, facilities utilize the same face mask vendors, so this recommendation is likely to be followed without problem.

The limitations of this meta-analysis not only relate to the applicability of its results but also offer insights for future studies. First, only two types of masks were evaluated. These two types were the most often compared in the literature, which facilitated the ability to apply meta-analysis to the data. Further mask types, such as cloth masks or face shields, are missing in the present meta-analysis. Furthermore, one study also investigated the combination of wearing several types of masks at the same time (e.g., N95 plus face shield) [22], which is also commonly used in medical practice [37,38,39]. Second, the majority of the nine included studies were found to be at moderate risk of bias (only two were at low risk of bias). Third, the signal processing is limited to the software Praat. Studies that used other software, such as the Multi Dimensional Voice Program or Analysis of Dysphonia in Speech and Voice from Pentax Medical or Dr. Speech, were excluded based on specification and reliability differences due to the application of different acoustic software packages. Fourth, the most evaluated voices were vocally healthy. A minority of voice-disordered voice samples were also included, but these types of voices reveal a high fluctuation and abnormality in the outcomes of the voice measures, which can have an influence on the variability of the measures for the present study. Fifth, the search strategy for relevant papers in this study was conducted in two languages, excluding other languages such as Asian languages, Spanish, or French that might have contained relevant publications. Sixth, the present meta-analysis evaluated mainly acoustic markers which are dedicated to voice quality. Other acoustic aspects for speech intelligibility, such as formant frequencies and spectral analysis, were not part of the present meta-analysis and have to be analyzed in the future as well. Seventh, the long-term effects of wearing masks will need to be studied in the future. There is some indication that the voice may change in acoustic voice quality markers after prolonged wearing of a mask evaluated in a longitudinal investigation over two years [40].

## 5. Conclusions

The present meta-analysis mainly included vocally healthy individuals without sufficient data from the clinical population because only two out of nine included studies considered participants with dysphonia. However, this study demonstrated no impact of RPMs on five acoustic voice quality parameters and SPL. However, mask type effects on acoustic parameters did differ significantly. In the current study, this was confirmed for Jit%, HNR, CPPS, and SPL. Thus, for clinical laryngology practice, it is recommended that if RPMs are used, then the same mask type should always be applied and not changed to keep the comparability and accuracy of the measurement results high.

## Figures and Tables

**Table 1:** Characteristics of cross-sectional trials in the meta-analysis.

Study	Sample Size	Voice Status	Age (Mean/Range in Years)/Gender	Types of Masks	Acoustic Parameters
Maggee et al. (2020) [15]	Total: *n* = 7	Vocally- healthy	28.1 (21–39) F/M = 3/4	No mask; surgical/medical mask; N95; Cloth mask	Jit%; Shim%; HNR; CPPS; SPL
Cavallaro et al. (2021) [16]	Total: *n* = 50	Vocally- healthy	47.0 (26–69) F/M = 30/20	No mask; surgical/medical mask	Jit%; Shim%; HNR
Nguyen et al. (2021) [17]	Total: *n* = 16	Vocally- healthy	43.0 (24–61) F/M = 12/4	No mask; surgical/medical mask; KN95;	HNR; CPPS; SPL
Lin et al. (2022) [18]	Total: *n* = 159	Vocally healthy (*n* = 53); VFBL (*n* = 59); IGC (*n* = 27); ESGC (*n* = 20)	Vocally healthy 42.62 (20–85) F/M = 28/25 Voice-disordered 47.7 (24–70) F/M = 49/57	No mask; surgical/medical mask	CPPS; SPL
Lehnert et al. (2022) [19]	Total: *n* = 31	Vocally healthy	Age unknown F/M = 18/13	No mask; surgical/medical mask; FFP2	AVQI
Fiorella et al. (2023) [20]	Total: *n* = 60	Vocally healthy	47.0 (26–69) F/M = 36/24	No mask; surgical/medical mask	Jit%; Shim%; HNR; SPL
Maryn et al. (2023) [21]	Total: *n* = 50	Vocally healthy (*n* = 12); VVD (*n* = 38)	44.9 (10–77) F/M = 29/21	No mask; surgical/medical mask; FFP2; transparent mask	Jit%; Shim%; HNR; CPPS; AVQI; SPL
Joshi et al. (In Press) [22]	Total: *n* = 19	Vocally- healthy	35.0 (18–67) F/M = 10/9	No mask; surgical/medical mask; KN95; Cloth mask; Face shield	CPPS; SPL
Gao et al. (In Press) [23]	Total: *n* = 30	Vocally- healthy	23.26 (20–40) F/M = 15/15	No mask; surgical/medical mask; N95	Jit%; Shim%; HNR; SPL

F: Female; M: Male; VFBL: vocal fold benign lesions; IGC: insufficient glottal closure; ESGC: early stage glottic carcinoma; VVD: various voice disorders; Jit%: jitter; Shim%: shimmer; HNR: harmonics-to-noise ratio; CPPS: cepstral peak prominence smooth; AVQI: Acoustic Voice Quality Index; SPL: sound pressure level, habitual sound intensity level.

**Table 2:** Meta-analysis by Treatment and by Voice Measures (Random Effects Model).

Comparison between Masks and Without	Parameter	*n*	Mean Difference (95% CI)	*p*-Value	I^2^	*p*-Value from Begg’s Test
Medical/Surgical masks vs. without	Jit%	197	−0.02% (−0.04% to 0.003%)	0.086	6.17%	0.573
	Shim%	197	−0.06% (−0.20% to 0.08%)	0.414	35.48%	0.851
	HNR	213	−0.17 dB (−0.69 dB to 0.35 dB)	0.522	0.22%	0.293
	CPPS	251	0.28 dB (−0.36 dB to 0.91 dB)	0.396	64.79%	0.174
	AVQI	81	0.06 dB (−0.47 dB to 0.60 dB)	0.824	71.58%	0.317
	SPL	341	−0.35 dB (−1.04 dB to 0.34 dB)	0.316	41.71%	0.152
FFP2/(K)N95 masks vs. without	Jit%	87	0.01% (−0.02% to 0.03%)	0.716	0.00%	0.602
	Shim%	87	−0.07% (−0.18% to 0.05%)	0.240	0.00%	0.602
	HNR	103	−1.37 dB (−2.79 dB to 0.05 dB)	0.059	59.48%	0.497
	CPPS	92	0.003 dB (−0.69 dB to 0.69 dB)	0.994	38.96%	0.050
	AVQI	81	0.05 (−0.70 to 0.79)	0.905	85.30%	0.317
	SPL	122	0.36 dB (−0.59 dB to 1.31 dB)	0.460	13.18%	0.348

Jit%: jitter; Shim%: shimmer; HNR: harmonics-to-noise ratio; CPPS: cepstral peak prominence smooth; AVQI: Acoustic Voice Quality Index; SPL: sound pressure level, habitual sound intensity level.

## Data Availability

The original contributions presented in the study are included in the article; further inquiries can be directed to the corresponding author.

## Supplementary Materials

The following supporting information can be downloaded at https://www.mdpi.com/article/10.3390/jcm12185922/s1, Table S1: Search strategies for systematic review; Table S2: Risk of Bias analyzed with AHRQ Methodology Checklist for cross-sectional studies. References [15,16,17,18,19,20,21,22,23] are cited in the Supplementary Materials.
