# Supplementary material for: The Impact of Protective Face Coverings on Acoustic Markers in Voice: A Systematic Review and Meta-Analysis
Source: J Clin Med. 2023 Sep 12;12(18):5922. doi: 10.3390/jcm12185922 (PMC10531748; doi:10.3390/jcm12185922)
Supplement: Supplementary file 1 [file jcm-12-05922-s001.zip › jcm-2585158-supplementary.pdf]

## Supplemental Table S1: Search strategies for systematic review

| Database | Search Strategy |
|---|---|
| MEDLINE via PubMed, CENTRAL via Cochrane Library, LIVIVO by ZB MED Search Portal for Life Sciences, Speechbite, and Google Scholar. | 1. (voice OR healthy voice OR vocal health OR voice impaired OR adults OR voice disorder OR vocal dysfunction OR dysphonia)<br>2. (Nose-and-Mouth-Covering respiratory protective mask OR protective mask OR mask OR face mask OR surgical mask OR face cover OR face shield OR face guard OR cloth mask OR N95 mask OR KN95 mask OR respiratory mask OR FFP2 mask)<br>3. (no mask OR different Nose-and Mouth-Covering respiratory protective mask OR different face mask)<br>4. (voice quality OR effect OR impact OR influence OR pitch OR intensity OR HNR OR AVQI OR CPPS OR Jitter OR Shimmer OR Praat)<br>5. (1 AND 2 AND 3 AND 4) |

## Supplemental Table S2: Risk of Bias analyzed with AHRQ Methodology Checklist for cross-sectional studies [15-23]

Magee et al. (2020) [15]

| Item | Yes | No | Unclear | Explanation |
|---|---|---|---|---|
| 1) Define the source of information (survey, record review) | X | | | Page 3563. Method section of Speech Acquisition and feature extraction for acoustic output |
| 2) List inclusion and exclusion criteria for exposed and unexposed subjects (cases and controls) or refer to previous publications | | | X | Page 3563. Participants were described (finally as vocally-healthy) but no clear inclusion and exclusion criteria were described. |
| 3) Indicate time period used for identifying patients | | X | | No clear time period mentioned |
| 4) Indicate whether or not subjects were consecutive if not population-based | | | X | This is assumed, but there is no precise description of this aspect in the article. |
| 5) Indicate if evaluators of subjective components of study were masked to other aspects of the status of the participants | | | X | N/A |
| 6) Describe any assessments undertaken for quality assurance purposes (e.g., test/retest of primary outcome measurements) | | X | | This aspect is not mentioned. |
| 7) Explain any patient exclusions from analysis | | | X | N/A |
| 8) Describe how confounding was assessed and/or controlled | X | | | Page 3564. Background noise controlled, standards by American Speech-Language-Hearing Association from 2018 of acoustic recordings were complied |
| 9) If applicable, explain how missing data were handled in the analysis | | | X | N/A |
| 10) Summarize patient response rates and completeness of data collection | X | | | Page 3564-3566. Result section |
| 11) Clarify what follow-up, if any, was expected and the percentage of patients for which incomplete data or follow-up was obtained | | X | | Page 3566-3567. Discussion section not further specified. |

➔ 50% moderate risk of bias

Cavallaro et al. (2021) [16]

| Item | Yes | No | Unclear | Explanation |
|---|---|---|---|---|
| 1) Define the source of information (survey, record review) | X | | | Page 2. Method section |
| 2) List inclusion and exclusion criteria for exposed and unexposed subjects (cases and controls) or refer to previous publications | X | | | Page 2. Method section |
| 3) Indicate time period used for identifying patients | | X | | No clear time period mentioned |
| 4) Indicate whether or not subjects were consecutive if not population-based | | | X | This is assumed, but there is no precise description of this aspect in the article. |
| 5) Indicate if evaluators of subjective components of study were masked to other aspects of the status of the participants | | | X | N/A |
| 6) Describe any assessments undertaken for quality assurance purposes (e.g., test/retest of primary outcome measurements) | | X | | This aspect is not mentioned. |
| 7) Explain any patient exclusions from analysis | | | X | N/A |
| 8) Describe how confounding was assessed and/or controlled | X | | | Page 2. Background noise controlled (<30dB), all subjects were trained to voice a vocal sample of a sustained /a/, at a conversational voice intensity, always within 55 dB and 65 dB, on average (not including recordings the average intensity of which was out of range) |
| 9) If applicable, explain how missing data were handled in the analysis | | | X | N/A |
| 10) Summarize patient response rates and completeness of data collection | X | | | Page 2-3. Result section |
| 11) Clarify what follow-up, if any, was expected and the percentage of patients for which incomplete data or follow-up was obtained | | X | | Page 2-4 Discussion section not further specified. |

➔ 57% = moderate risk of bias

Nguyen et al. (2021) [17]

| Item | Yes | No | Unclear | Explanation |
|---|---|---|---|---|
| 1) Define the source of information (survey, record review) | X | | | Page 3. Method section of voice recordings |
| 2) List inclusion and exclusion criteria for exposed and unexposed subjects (cases and controls) or refer to previous publications | | | X | Page 2. Participants were described (finally as vocally-healthy) but no clear inclusion and exclusion criteria were described. |
| 3) Indicate time period used for identifying patients | | X | | No clear time period mentioned |
| 4) Indicate whether or not subjects were consecutive if not population-based | | | X | This is assumed, but there is no precise description of this aspect in the article. |
| 5) Indicate if evaluators of subjective components of study were masked to other aspects of the status of the participants | | | X | N/A |
| 6) Describe any assessments undertaken for quality assurance purposes (e.g., test/retest of primary outcome measurements) | X | | | Page 4. Quality check of voice recordings and reliability analysis |
| 7) Explain any patient exclusions from analysis | | | X | N/A |
| 8) Describe how confounding was assessed and/or controlled | X | | | Page 3. Method section of voice recordings (randomized order of conditions, standards by American Speech-Language-Hearing Association from 2018 of acoustic recordings were complied) |
| 9) If applicable, explain how missing data were handled in the analysis | | | X | N/A |
| 10) Summarize patient response rates and completeness of data collection | X | | | Page 5-7. Result section |
| 11) Clarify what follow-up, if any, was expected and the percentage of patients for which incomplete data or follow-up was obtained | X | | | Page 7-8. Discussion section |

- ➔ 83% = low risk of bias

Lin et al. (2022) [18]

| Item | Yes | No | Unclear | Explanation |
|---|---|---|---|---|
| 1) Define the source of information (survey, record review) | X | | | Page 1744. Method section |
| 2) List inclusion and exclusion criteria for exposed and unexposed subjects (cases and controls) or refer to previous publications | X | | | Page 1743. Method section |
| 3) Indicate time period used for identifying patients | X | | | Page 1743. Method section |
| 4) Indicate whether or not subjects were consecutive if not population-based | X | | | Page 1743. Method section |
| 5) Indicate if evaluators of subjective components of study were masked to other aspects of the status of the participants | | X | | Page 1744. Samples are mixed but not masked. |
| 6) Describe any assessments undertaken for quality assurance purposes (e.g., test/retest of primary outcome measurements) | | X | | This aspect is not mentioned. |
| 7) Explain any patient exclusions from analysis | X | | | Page 1743. Method section |
| 8) Describe how confounding was assessed and/or controlled | | X | | Page 1744. standards by American Speech-Language-Hearing Association from 2018 of acoustic recordings were not complied (e.g., recordings saved as MP3 format, microphone) |
| 9) If applicable, explain how missing data were handled in the analysis | | | X | N/A |
| 10) Summarize patient response rates and completeness of data collection | X | | | Page 1744-1747. Result section |
| 11) Clarify what follow-up, if any, was expected and the percentage of patients for which incomplete data or follow-up was obtained | X | | | Page 1748. Discussion section. |

➔ 70% = moderate risk of bias

Lehnert et al. (2022) [19]

| Item | Yes | No | Unclear | Explanation |
|---|---|---|---|---|
| 1) Define the source of information (survey, record review) | X | | | Page 4618. Method section |
| 2) List inclusion and exclusion criteria for exposed and unexposed subjects (cases and controls) or refer to previous publications | | | X | Page 4618. Participants were less described (finally as vocally-healthy) but no clear inclusion and exclusion criteria were described. |
| 3) Indicate time period used for identifying patients | | X | | This aspect is not mentioned. |
| 4) Indicate whether or not subjects were consecutive if not population-based | | | X | This is assumed, but there is no precise description of this aspect in the article. |
| 5) Indicate if evaluators of subjective components of study were masked to other aspects of the status of the participants | | | X | N/A |
| 6) Describe any assessments undertaken for quality assurance purposes (e.g., test/retest of primary outcome measurements) | | X | | This aspect is not mentioned. |
| 7) Explain any patient exclusions from analysis | | X | | This aspect is not mentioned. |
| 8) Describe how confounding was assessed and/or controlled | X | | | Page 4618. Randomized order, standards by American Speech-Language-Hearing Association from 2018 of acoustic recordings were complied, |
| 9) If applicable, explain how missing data were handled in the analysis | | | X | N/A |
| 10) Summarize patient response rates and completeness of data collection | X | | | Page 4618-4620. Result section |
| 11) Clarify what follow-up, if any, was expected and the percentage of patients for which incomplete data or follow-up was obtained | X | | | Page 4620. Discussion section. |

➔ 57% = moderate risk of bias

Fiorella et al. (2023) [20]

| Item | Yes | No | Unclear | Explanation |
|---|---|---|---|---|
| 1) Define the source of information (survey, record review) | X | | | Page 467.e2. Method section |
| 2) List inclusion and exclusion criteria for exposed and unexposed subjects (cases and controls) or refer to previous publications | X | | | Page 467.e2. Method section |
| 3) Indicate time period used for identifying patients | | X | | This aspect is not mentioned. |
| 4) Indicate whether or not subjects were consecutive if not population-based | | | X | This is assumed, but there is no precise description of this aspect in the article. |
| 5) Indicate if evaluators of subjective components of study were masked to other aspects of the status of the participants | | | X | N/A |
| 6) Describe any assessments undertaken for quality assurance purposes (e.g., test/retest of primary outcome measurements) | | X | | This aspect is not mentioned. |
| 7) Explain any patient exclusions from analysis | | X | | This aspect is not mentioned. |
| 8) Describe how confounding was assessed and/or controlled | X | | | Page 467.e2. Calibration procedure of the recording system, standards by American Speech-Language-Hearing Association from 2018 of acoustic recordings were complied, background noise < 30 dB |
| 9) If applicable, explain how missing data were handled in the analysis | | | X | N/A |
| 10) Summarize patient response rates and completeness of data collection | X | | | Page 467.e3. Result section |
| 11) Clarify what follow-up, if any, was expected and the percentage of patients for which incomplete data or follow-up was obtained | | X | | This aspect is minor mentioned. |

- ➔ 50%= moderate risk of bias

Maryn et al. (2023) [21]

| Item | Yes | No | Unclear | Explanation |
|---|---|---|---|---|
| 1) Define the source of information (survey, record review) | X | | | Page 468.e2. Method section |
| 2) List inclusion and exclusion criteria for exposed and unexposed subjects (cases and controls) or refer to previous publications | | | X | This aspect is not clear mentioned. |
| 3) Indicate time period used for identifying patients | | X | | This aspect is not mentioned; also not in the reference. |
| 4) Indicate whether or not subjects were consecutive if not population-based | X | | | Page 468.e2. Method section |
| 5) Indicate if evaluators of subjective components of study were masked to other aspects of the status of the participants | | | X | N/A |
| 6) Describe any assessments undertaken for quality assurance purposes (e.g., test/retest of primary outcome measurements) | X | | | Page 468.e2-468.e4. Method section |
| 7) Explain any patient exclusions from analysis | | | X | N/A |
| 8) Describe how confounding was assessed and/or controlled | X | | | Page 468.e2-468.e4. Method section |
| 9) If applicable, explain how missing data were handled in the analysis | | | X | N/A |
| 10) Summarize patient response rates and completeness of data collection | X | | | Page 468.e4-468.e6. Result section |
| 11) Clarify what follow-up, if any, was expected and the percentage of patients for which incomplete data or follow-up was obtained | X | | | Page 468.e9. Result section |

➔ 86% = low risk of bias

Joshi et al. (In Press) [22]

| Item | Yes | No | Unclear | Explanation |
|---|---|---|---|---|
| 1) Define the source of information (survey, record review) | X | | | Page 3. Method section |
| 2) List inclusion and exclusion criteria for exposed and unexposed subjects (cases and controls) or refer to previous publications | | | X | Page 2. Participants were described (finally as vocally-healthy) but no clear inclusion and exclusion criteria were described. |
| 3) Indicate time period used for identifying patients | | X | | This aspect is not mentioned. |
| 4) Indicate whether or not subjects were consecutive if not population-based | | | X | This is assumed, but there is no precise description of this aspect in the article. |
| 5) Indicate if evaluators of subjective components of study were masked to other aspects of the status of the participants | | | X | N/A |
| 6) Describe any assessments undertaken for quality assurance purposes (e.g., test/retest of primary outcome measurements) | | X | | This aspect is not mentioned. |
| 7) Explain any patient exclusions from analysis | | | X | N/A |
| 8) Describe how confounding was assessed and/or controlled | | X | | Page 3. standards by American Speech-Language-Hearing Association from 2018 of acoustic recordings were not complied (e.g., microphone, high SPL background noise < 60dB) |
| 9) If applicable, explain how missing data were handled in the analysis | | | X | N/A |
| 10) Summarize patient response rates and completeness of data collection | X | | | Page 3&4. Result section |
| 11) Clarify what follow-up, if any, was expected and the percentage of patients for which incomplete data or follow-up was obtained | X | | | Page 7. Discussion section |

➔ 50% = moderate risk of bias

Gao et al. (In Press) [23]

| Item | Yes | No | Unclear | Explanation |
|---|---|---|---|---|
| 1) Define the source of information (survey, record review) | X | | | Page 2. Method section |
| 2) List inclusion and exclusion criteria for exposed and unexposed subjects (cases and controls) or refer to previous publications | | | X | Page 2. Participants were described (finally as vocally-healthy) but no clear inclusion and exclusion criteria were described. |
| 3) Indicate time period used for identifying patients | | X | | This aspect is not mentioned. |
| 4) Indicate whether or not subjects were consecutive if not population-based | | | X | This is assumed, but there is no precise description of this aspect in the article. |
| 5) Indicate if evaluators of subjective components of study were masked to other aspects of the status of the participants | | | X | N/A |
| 6) Describe any assessments undertaken for quality assurance purposes (e.g., test/retest of primary outcome measurements) | | X | | This aspect is not mentioned. |
| 7) Explain any patient exclusions from analysis | | | X | N/A |
| 8) Describe how confounding was assessed and/or controlled | X | | | Page 2. Latin square counterbalancing order, standards by American Speech-Language-Hearing Association from 2018 of acoustic recordings were complied (e.g., microphone, low background noise <30 dB) |
| 9) If applicable, explain how missing data were handled in the analysis | | | X | N/A |
| 10) Summarize patient response rates and completeness of data collection | X | | | Page 3-6. Result section |
| 11) Clarify what follow-up, if any, was expected and the percentage of patients for which incomplete data or follow-up was obtained | | X | | This aspect is not mentioned. |

➔ 50% = moderate risk of bias
